# Crystal structure of bis­{2-hy­droxy-*N*′-[1-(pyrazin-2-yl)ethyl­idene]benzohydrazidato}cadmium(II)

**DOI:** 10.1107/S2056989021000657

**Published:** 2021-01-22

**Authors:** Ping Yang, Xiao-Bao Xie, Qing-Shan Shi

**Affiliations:** a Guangdong Provincial Key Laboratory of Microbial Culture Collection and Application, State Key Laboratory of Applied Microbiology Southern China, Guangdong Institute of Microbiology, Guangdong Academy of Sciences, Guangzhou 510070, People’s Republic of China

**Keywords:** crystal structure, cadmium(II) complex, hydrazone derivatives

## Abstract

In the title complex mol­ecule, [Cd(C_13_H_11_N_4_O_2_)_2_], the Cd atom is coordinated in a distorted octa­hedral geometry by two tridentate ligands synthesized from 2-hy­droxy­benzohydrazide and 1-(pyrazin-2-yl)ethan-1-one. The mol­ecule has twofold crystallographic symmetry and is isomorphous to its Mn, Co, Ni, Cu and Zn counterparts.

## Abstract 2

The structure of a cadmium(II) aroylhydrazone complex, *viz*. bis­{2-hy­droxy-*N*′-[1-(pyrazin-2-yl)ethyl­idene]benzohydrazidato}cadmium(II) is described.

## Chemical context   

Aroylhydrazones are competent ligands for various functional coordination compounds. They have the ability of polydentate coordination and are often used as building units of polynuclear magnetic compounds (Huang *et al.*, 2016 ▸; Zhang *et al.*, 2010 ▸). Aroylhydrazones can exhibit keto–enol tautomerism, and the uncomplexed aroylhydrazone ligand is commonly found in its keto form (Kalinowski *et al.*, 2008 ▸; Tai & Feng, 2008 ▸). Metal complexes of deprotonated aroylhydrazones have been used in various catalytic and biological applications (Sutradhar *et al.*, 2013 ▸; Yang *et al.*, 2019 ▸; Yang, Chen *et al.*, 2020 ▸). Aroylhydrazones synthesized from aryl­hydrazides and aromatic aldehydes/ketones with a nitro­gen or oxygen atom in the *ortho* position can coordinate to metals in a tridentate chelating mode (Cindrić *et al.*, 2017 ▸; Patel *et al.*, 2018 ▸; You *et al.*, 2018 ▸), and they have been used as probes and chemosensors for various metal ions. For example, the aroylhydrazone ligand containing a 4-(di­methyl­amino)­phenylprop­enyl or benzamide substituent specifically senses Al^3+^, Cd^2+^ (Kar *et al.*, 2015 ▸) and Ni^2+^ ions (Manna *et al.*, 2019 ▸) through significant changes in their absorption and emission spectroscopic behaviour after complexation with the metal ions. Here, we study the coord­ination attributes of an aroylhydrazone with cadmium.
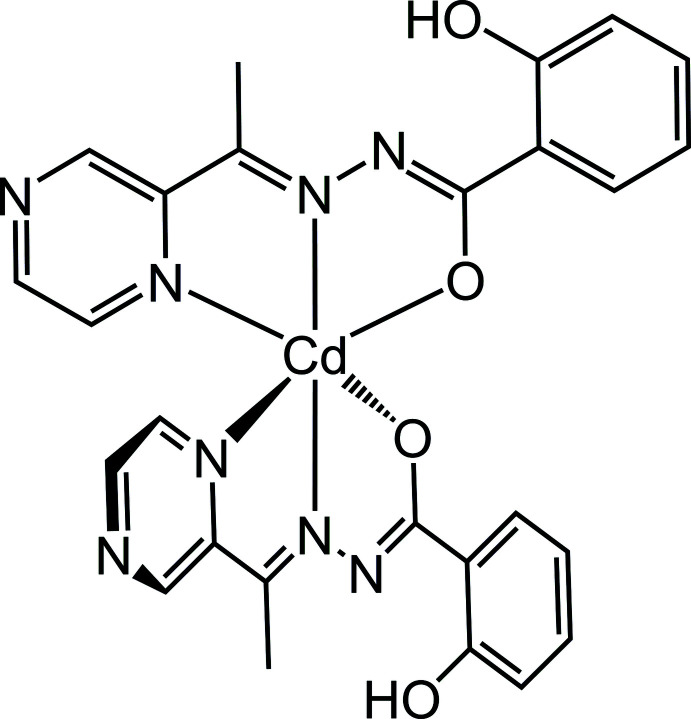


## Structural commentary   

**Figure 1:**
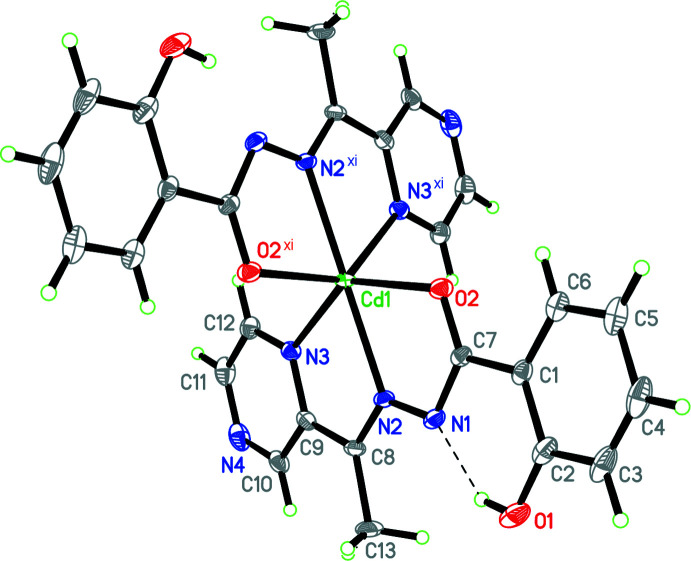
The mol­ecular structure of [Cd(C_13_H_11_N_4_O_2_)_2_] with displacement ellipsoids at the 30% probability level. Symmetry code: (xi) −*x* + 1, −*y* + 1, *z*.

In the title complex, the Cd^2+^ ion possesses a distorted octa­hedral N_4_O_2_ coordination environment, which is generated by the two deprotonated ligands *L* (Fig. 1 ▸). The complex is bis­ected by a twofold crystallographic axis with the two ligands being equivalent by crystal symmetry. The complex is isomorphous to its Mn, Co, Ni, Cu and Zn counterparts (Yang *et al.*, 2019 ▸; Yang, Zhang *et al.*, 2020 ▸). The O2—C7 and C7—N1 bond lengths in the title compound are 1.255 (5) Å and 1.355 (5) Å, respectively, indicating that the coordinated ligands are closer to the keto than the enol form, but are slightly more delocalized than in the purely keto tautomeric form as found in the free ligand form of similar aroylhydrazones. The free ligand *L* has not yet been structurally described, but the equivalent bond distances in *e.g*. 2-hy­droxy-*N*′-[1-(3-methyl­pyrazin-2-yl)ethyl­idene]benzohydrazide, *L* ^1^, with one more methyl group on pyrazine (Tai & Feng, 2008 ▸), were reported as 1.235 and 1.340 Å, respectively.

The ligand in the title complex is close to planar (the mean deviation from the average plane is 0.0763 Å). The largest deviation from planarity is only 0.145 (3) Å, observed for atom C12 of the pyrazine ring. The Cd1 atom is nearly coplanar with each of the two ligands (deviation = 0.316 Å). The dihedral angle between the two ligands is 78.705 (16)°. The oxygen atom O1 of the phenolic group remains protonated, and forms an intra­molecular hydrogen bond O1—H1⋯N1 [2.557 (4) Å, 146 (7)°].

The intra­molecular hydrogen bond stabilizes the planar geometry of the ligand. The presence of the intra­molecular hydrogen bond does also appear to affect the propensity of the metal complex towards crystallization. We found that when the hydroxyl group is in the *meta* or *para* position {3-hy­droxy-*N*′-[1-(pyrazin-2-yl)ethyl­idene]benzohydrazide (*L* ^2^) or 4-hy­droxy-*N*′-[1-(pyrazin-2-yl)ethyl­idene]benzohydrazide (*L* ^3^)}, where no intra­molecular hydrogen bond can be formed, crystallization is substanti­ally delayed and a much longer time is required for the complexes to crystallize.

In the isomorphous Mn, Co, Ni, Cu and Zn *M*(*L*)_2_ complexes, the ligands are also close to planar (the mean deviation from the average plane ranges from 0.0608 to 0.0754 Å). In di­methyl­formamide (DMF)-solvated Ni and Cu complexes of similar ligands *L* ^2^ {3-hy­droxy-*N*′-[1-(pyrazin-2-yl)ethyl­idene]benzohydrazide} and *L* ^3^ {4-hy­droxy-*N*′-[1-(pyr­az­in-2-yl)ethyl­idene]benzohydrazide} [*M*(*L* ^2^)_2_]·2(DMF) (*M* = Ni, Cu and Zn) and [Cu(*L* ^3^)_2_]·2(DMF) (*M* = Ni and Cu), the planarity of the ligands is reduced, with a mean deviation from the average plane between 0.2164 to 0.2290 Å.

In the title complex, the Cd1—N3, Cd1—N2 and Cd1—O2, bond lengths are 2.356 (3), 2.273 (3) and 2.277 (4) Å, respectively, which are close to typical for Cd^2+^ complexes closely related to the title compound, such as bis­{*N*′-[1-(pyridin-2-yl)ethyl­idene]benzohydrazidato}cadmium(II) (Sen *et al.*, 2005 ▸), bis­{2-[2-(pyridin-2-yl­methyl­ene)hydrazine-1-carbon­yl]benzene­sulfonamide}­cadmium(II) (Sousa-Pedrares *et al.*, 2008 ▸) and bis­[*N*′-(2-hy­droxy­benzo­yl)picolino­hydra­zon­am­ide]­cadmium(II) (Xu *et al.*, 2014 ▸), bis­{*N*′-[di(pyridin-2-yl)methyl­ene]benzohydrazidato}cadmium(II) (Kuriakose *et al.*, 2017 ▸) [the range of N—Cd is 2.360 (12)–2.4135 (11) Å, N(middle)—Cd 2.225 (2)–2.295 (2) Å, O—Cd 2.240 (2)–2.358 (10) Å].

The coordination environment of the Cd ion is highly distorted octa­hedral, caused by the rigidity of the ligand and its small N—N and N—O bite angles of only 69.86 (11) (N3—Cd1—N2) and 69.83 (11)° (N2—Cd1—O2). As a result, the N—Cd—O, N–Cd—N and O—Cd—O angles in the title compound deviate substanti­ally from the values of 180 and 90° expected for an idealized octa­hedral complex. The *trans* angles range from 139.07 (10) to 170.63 (17)°, while the *cis* angles vary between 69.83 (11) and 117.27 (11)°.

Bond distances and angles within the isomorphous series of the Mn, Co, Ni, Cu, Zn, and Cd complexes follow a trend consistent with the metal ion radius (Table 1 ▸). Bond lengths first decrease and then increase, with a minimum value for the Ni or Cu complexes, and a maximum for the title cadmium complex as a result of its substanti­ally larger ion radius as the only 4*d* complex of the series. The trend of the N—*M*—O angle (within the same ligand) is opposite to that of the metal ion radius, and first increases and then decreases, with the maximum value appearing for the Ni complex (Brines *et al.*, 2007 ▸; Reger *et al.*, 2012 ▸; Sola *et al.*, 1994 ▸; Database of Ionic Radii, 2020 ▸). The distortion from octa­hedral geometry increases with ion radius, and is most pronounced for the title cadmium complex, as can be seen for *e.g*. the N(mid)—*M*—N(mid) angles, which range from 172.30 to 174.46° for the 3*d* complexes, while the value for the 4*d* Cd complex is 170.63 (17)°.

## Supra­molecular features   

**Figure 2:**
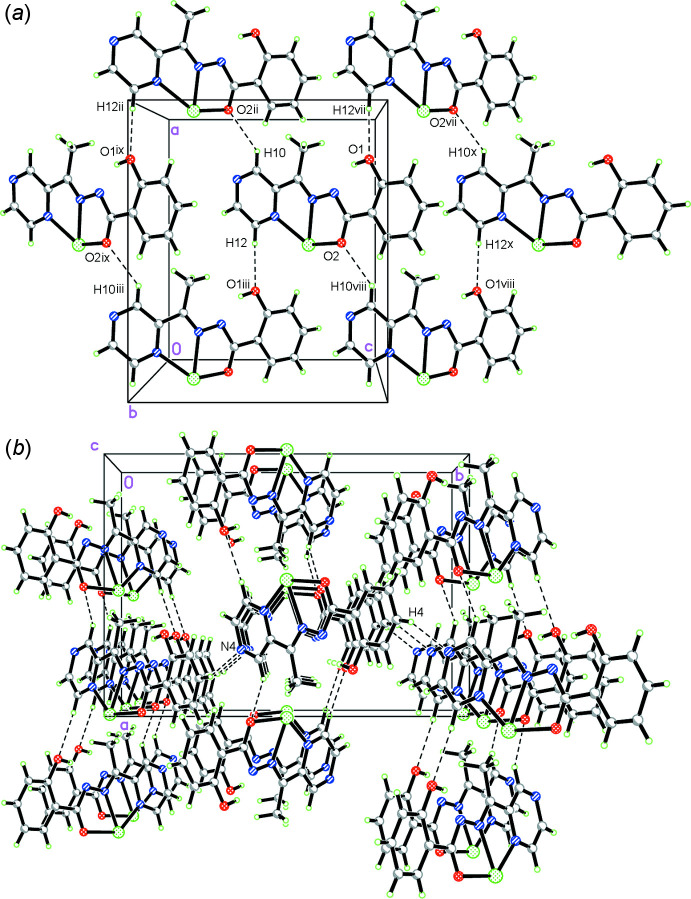
Crystal packing of the title compound showing the O—H⋯O and C—H⋯N hydrogen bonds. For clarity, another symmetrical ligand coordinated with metal center has been omitted. Symmetry codes: (ii) *x* + 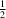, −*y* + 1, *z* − 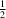; (iii) *x* − 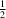, −*y* + 1, *z* − 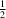; (vii) *x* + 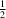, −*y* + 1, *z* + 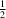; (viii) *x* − 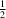, −*y* + 1, *z* + 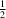; (ix) *x*, *y*, *z* − 1; (*x*) *x*, *y*, *z* + 1.

**Figure 3:**
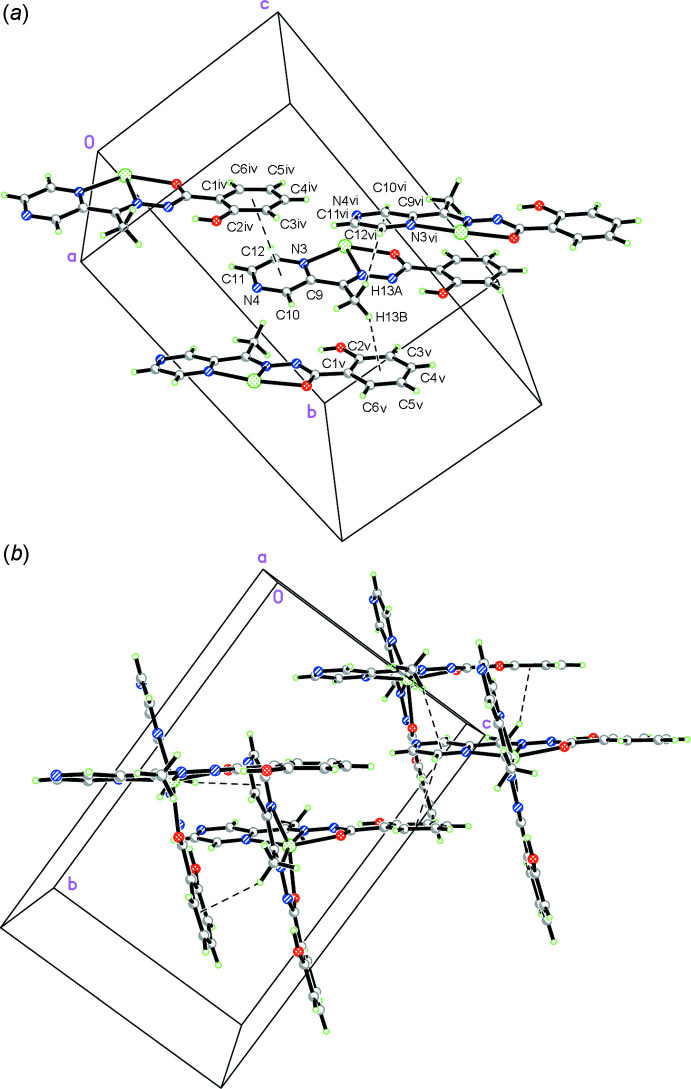
(*a*) Crystal packing of the title compound showing the C—H⋯π and π–π inter­actions. For clarity, the second ligand at each metal centre has been omitted. Symmetry codes: (iv) *x*, *y* − 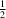, *z* − 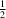; (v) −*x* + 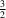, *y*, *z* − 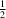; (vi) −*x* + 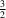, *y*, *z* + 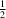. (*b*) View down [100].

Two types of weak inter­molecular inter­actions, C—H⋯N and C—H⋯O hydrogen bonds and π–π stacking and C—H⋯π inter­actions, have a significant impact on the packing of the complexes in the solid state. Three inter­molecular hydrogen bonds (Table 2 ▸) are observed in the crystal. Two hydrogen bonds (C10—H10⋯O2^ii^ and C12—H12⋯O1^i^, symmetry code given in Table 2 ▸; Fig. 2 ▸ *a*) form a sheet parallel to the crystallographic *bc* plane. Adjacent sheets of the complex are connected to each other *via* a weak C4—H4⋯N4^iii^ inter­action, forming a three-dimensional network (Table 2 ▸ and Fig. 2 ▸ *b*). Inter­molecular π–π stacking is observed between the pyrazine rings and benzene rings of ligands in neighbouring complexes [the centroid–centroid distance between N3–N4/C9–C12 and C1–C6^vi^ [symmetry code: (vi) *x*, *y* − 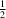, *z* − 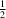] is 3.641 (2) Å, with a slippage of 1.252 Å, Fig. 3 ▸. Inter­molecular inter­actions between carbon atoms C13 and the π ring of lateral benzene rings and pyrazine rings in neighbouring mol­ecules are found, namely C13—H13*B*⋯*Cg*1^v^ [2.71 Å, *Cg*1 is the centroid of the C1–C6 ring; symmetry code: (v) −*x* + 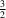, *y*, *z* − 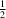] and C13–H13*A*⋯*Cg*2^iv^ [2.86 Å, *Cg*2 is the centroid of the N3–N4/C9–C12; symmetry code: (iv) −*x* + 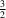, *y*, *z* + 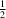] (Fig. 3 ▸).

## Database survey   

A search of the Cambridge Structural Database (CSD, version 5.41, August 2020; Groom *et al.*, 2016 ▸) for metal complexes involving the *N*′-[1-(pyrazin-2-yl)ethyl­idene]benzohydrazide ligand resulted in seven related metal complexes with exactly the same ligand. These are the already discussed isomorphous Mn, Co, Ni, Cu and Zn [*M* ^II^(*L*)_2_] complexes (CCDC refcodes: CIZJED for *M* = Mn, CIZGOK for *M* = Co, CIZGAW for *M* = Ni (Yang, Zhang *et al.*, 2020 ▸), COYVUK for *M* = Cu and CIZFUP for *M* = Zn) (Yang *et al.*, 2019 ▸). In all of these complexes, the ligand *L* acts as a tridentate chelating ligand to generate a distorted octa­hedral structure with a close to planar ligand. Several complexes of related ligands have been found to be also isomorphous to the above series, crystallizing in the same *Aba*2 space group. These are a Co and a Zn complex bearing the ligand *N*′-[1-(pyrazin-2-yl)ethyl­idene]benzohydrazide (*L* ^4^, with one less hydroxyl group on benzene) (YELKUY, YELWUK; Tai *et al.*, 2008 ▸) as well as four metal complexes involving the ligand 2-hy­droxy-*N*′-[1-(pyridin-2-yl)ethyl­idene]benzohydrazide (*L* ^5^, substituted pyrazine group with pyridine group) with *M* = Cu^2+^, Ni^2+^, Zn^2+^ and Fe^2+^ [ADEYAK (Dang *et al.*, 2006*a* ▸); XENFEC (Dang *et al.*, 2006*b* ▸); HIGPOD (Barbazán *et al.*, 2007 ▸); RADDOR (Zhang *et al.*, 2010 ▸)].

There are also complexes of ligands *L* ^4^ and *L* ^5^ that are not isomorphous to the title complex: complex Cu_2_(*L* ^4^)_2_Cl_2_ is binuclear, where each Cu centre has two μ-chlorine ligands along with a tridentate coordinated *L* ^4^ mol­ecule, giving rise to a distorted square-pyramidal coordination environment. It belongs to the triclinic *P* 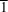 space group (YELXAR; Tai *et al.*, 2008 ▸). The cobalt complex [Co(*L* ^5^)_2_(ClO_4_)]·0.25(CH_3_OH) (IGAZAS; Shit *et al.*, 2009 ▸) has a nearly ideal octa­hedral structure in the monoclinic *P*2_1_/*n* space group, and the ligands have N—N and N—O bite angles of 81.70 to 83.11°. Cu(*L* ^5^)Br (HIGPIX; Barbazán *et al.*, 2007 ▸) and Cu(*L* ^5^)(NO_3_) (YILYEY; You *et al.*, 2018 ▸) have roughly square-planar coordination geometries. [Sb(*L* ^5^)Cl_2_]·H_2_O (YILYEY; Abboud *et al.*, 2007 ▸) has a square-pyramidal coordination geometry in the monoclinic *P*2_1_/*n* space group. Cu_2_(*L* ^5^)_2_Cl_2_ (NICYOP; Mondal *et al.*, 2013 ▸) is a binuclear complex and each Cu centre has a square-pyramidal coordination geometry. It is isomorphic to Cu_2_(*L* ^4^)_2_Cl_2_. A Zn complex, Zn(*L* ^1^)_2_·H_2_O (XIYNUP; Tai *et al.*, 2008 ▸) with the ligand *L* ^1^ with one more methyl group on pyrazine crystallizes in the monoclinic *P*2_1_/*n* space group. The planarity of the ligand is decreased compared to the title complex, and the Zn ion exhibits a distorted octa­hedral geometry. Also reported are five similar compounds featuring the ligands *L* ^2^ and *L* ^3^ with the hydroxyl group in the *meta* and *para* positions of the benzene ring, respectively. They crystallize as DMF solvates [*M*(*L* ^2^)_2_]·2(DMF) (DMF = di­methyl­formamide; *M* = Ni, Cu and Zn; CIZHIF, CIZGUQ and CIZJAZ) and [Cu(*L^3^*)_2_]·2(DMF) (*M* = Ni and Cu; CIZHUR and COYWEV) (Yang *et al.*, 2019 ▸; Yang, Zhang *et al.*, 2020 ▸) in the ortho­rhom­bic *Pbcn* space group. They also feature distorted octa­hedral structures and the planarity of the ligands is decreased compared to the title compound. All the complexes with the [*M*(Ligand)_2_] core are distorted octa­hedral, and all metal centres have a *mer* geometry. All ligands *L*, *L* ^1^, *L* ^2^, *L* ^3^, *L* ^4^ and *L* ^5^ are tridentate chelating.

## Synthesis and crystallization   

The title complex and ligand were synthesized according to literature procedures (Yang, Zhang *et al.*, 2020 ▸; Yang *et al.*, 2019 ▸). The complex was obtained by mixing a solution of the aroylhydrazone (0.02 mmol) in methanol (2 mL) and a solution of Cd(NO_3_)_2_·4H_2_O (0.01 mmol) in water (2 mL). After two weeks of static volatilization in a test tube at room temperature, clear light-yellow block-shaped crystals of Cd(*L*)_2_ were obtained (5.6 mg, yield 90%) (calculated based on metal ions), m.p. > 543 K. IR (KBr): ν (cm^−1^) = 1594 *s*, 1534 *s*, 1518 *s*, 1489 *s*, 1458 *s*, 1401 *w*, 1349 *s*, 1299 *s*, 1248 *m*, 1225 *m*, 1198 *m*, 1162 *m*, 1147 *s*, 1106 *w*, 1072 *s*, 1042 *m*, 1029 *m*, 910 *w*, 850 *w*, 833 *w*, 786 *w*, 764 *m*, 701 *m*, 662 *w*, 565 *w*, 541 *w*, 492 *w*, 419 *w*, 406 *w*.

## Refinement   

Crystal data, data collection and structure refinement details are summarized in Table 3 ▸. All C-bound H atoms were placed in calculated positions (C*sp* ^2^—H = 0.95 Å and C*sp* ^3^—H = 0.98 Å) and were included in the refinement in a riding-model approximation, with *U*_iso_(H) set to 1.2*U*_eq_(C*sp* ^2^) and 1.5*U*_eq_(C*sp* ^3^). The O-bound H atom was located based on a difference-Fourier map and its position was freely refined. It was assigned *U*_iso_(H) = 1.5*U*_eq_(O).

## Supplementary Material

Crystal structure: contains datablock(s) I. DOI: 10.1107/S2056989021000657/zl5004sup1.cif

Structure factors: contains datablock(s) I. DOI: 10.1107/S2056989021000657/zl5004Isup2.hkl

CCDC reference: 2051612

Additional supporting information:  crystallographic information; 3D view; checkCIF report

## Figures and Tables

**Table 1:** Comparative analysis of ion radius and the bond lengths and bond angles of coordination polyhedra (Å, °)

	Mn^2+^	Co^2+^	Ni^2+^	Cu^2+^	Zn^2+^	Cd^2+^
Ion radius	0.83	0.745	0.69	0.73	0.74	0.95
*M*—N	2.283	2.151	2.114	2.192	2.215	2.356
*M*—N(mid)	2.193	2.050	1.994	1.979	2.074	2.273
*M*—O	2.148	2.102	2.097	2.130	2.125	2.277
N(mid)—*M*—N(mid)	174.46	172.30	173.86	173.64	173.97	170.63
N—*M*—O (within the same ligand)	142.08	148.53	153.95	151.98	148.84	139.07
O—*M*—O	99.76	102.86	95.26	97.43	98.87	95.39
N(mid)—*M*—O (within different ligands)	104.25	99.33	98.33	98.79	100.49	103.58
CSD refcode	CIZJED*^*a*^*	CIZGOK*^*b*^*	CIZGAW*^*c*^*	COYVUK*^*d*^*	CIZFUP*^*e*^*	2051612*^*f*^*

Notes: (*a*) Yang (2019 ▸); (*b*) Yang (2019 ▸); (*c*) Yang, Zhang *et al.* (2020 ▸); (*d*) Yang, Zhang *et al.* (2019 ▸); (*e*) Yang (2019 ▸); (*f*) this work.

**Table 2:** Hydrogen-bond geometry (Å, °) *Cg*1 and *Cg*2 are the centroids of the C1–C6 and N3–N4/C9–C12 rings, respectively.

*D*—H⋯*A*	*D*—H	H⋯*A*	*D*⋯*A*	*D*—H⋯*A*
C4—H4⋯N4^i^	0.95	2.47	3.349 (6)	154
C10—H10⋯O2^ii^	0.95	2.55	3.283 (5)	134
C12—H12⋯O1^iii^	0.95	2.49	3.439 (5)	174
O1—H1⋯N1	0.90 (8)	1.76 (8)	2.557 (4)	146 (7)
C13—H13*A*⋯*Cg*2^iv^	0.98	2.86	3.740 (6)	149
C13—H13*B*⋯*Cg*1^v^	0.98	2.71	3.592 (6)	150

Symmetry codes: (i) 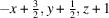; (ii) 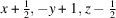; (iii) 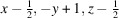; (iv) 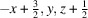; (v) 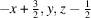.

**Table 3:** Experimental details

Crystal data	
Chemical formula	[Cd(C_13_H_11_N_4_O_2_)_2_]
*M*_r_	622.91
Crystal system, space group	Orthorhombic, *A* *b* *a*2
Temperature (K)	108
*a*, *b*, *c* (Å)	12.6654 (1), 17.63940 (18), 10.88800 (11)
*V* (Å^3^)	2432.49 (4)
*Z*	4
Radiation type	Cu *K*α
μ (mm^−1^)	7.64
Crystal size (mm)	0.12 × 0.10 × 0.08

Data collection	
Diffractometer	Rigaku Oxford Diffraction XtaLAB Synergy R, DW system, HyPix
Absorption correction	Multi-scan (*CrysAlis PRO*; Rigaku OD, 2020 ▸)
*T*_min_, *T*_max_	0.519, 1.000
No. of measured, independent and observed [*I* > 2σ(*I*)] reflections	36223, 2475, 2458
*R*_int_	0.038
(sin θ/λ)_max_ (Å^−1^)	0.630

Refinement	
*R*[*F* ^2^ > 2σ(*F* ^2^)], *wR*(*F* ^2^), *S*	0.026, 0.075, 1.20
No. of reflections	2475
No. of parameters	182
No. of restraints	1
H-atom treatment	H atoms treated by a mixture of independent and constrained refinement
Δρ_max_, Δρ_min_ (e Å^−3^)	0.72, −0.99
Absolute structure	Flack *x* determined using 1131 quotients [(*I* ^+^)−(*I* ^−^)]/[(*I* ^+^)+(*I* ^−^)] (Parsons *et al.*, 2013 ▸).
Absolute structure parameter	−0.012 (4)

Computer programs: *CrysAlis PRO* (Rigaku OD, 2020 ▸), *SHELXS* and *SHELXP* (Sheldrick, 2008 ▸), *SHELXL* (Sheldrick, 2015 ▸) and *publCIF* (Westrip, 2010 ▸).

## supplementary crystallographic information

### Crystal data

**Table:**

[Cd(C_13_H_11_N_4_O_2_)_2_]	*D*_x_ = 1.701 Mg m^−^^3^
*M**_r_* = 622.91	Cu *K*α radiation, λ = 1.54178 Å
Orthorhombic, *A**b**a*2	Cell parameters from 31744 reflections
*a* = 12.6654 (1) Å	θ = 2.5–76.5°
*b* = 17.63940 (18) Å	µ = 7.64 mm^−^^1^
*c* = 10.88800 (11) Å	*T* = 108 K
*V* = 2432.49 (4) Å^3^	Block, clear light yellow
*Z* = 4	0.12 × 0.10 × 0.08 mm
*F*(000) = 1256	

### Data collection

**Table:**

Rigaku Oxford Diffraction XtaLAB Synergy R, DW system, HyPix diffractometer	2475 independent reflections
Radiation source: Rotating-anode X-ray tube	2458 reflections with *I* > 2σ(*I*)
Detector resolution: 10.0000 pixels mm^-1^	*R*_int_ = 0.038
ω scans	θ_max_ = 76.2°, θ_min_ = 5.0°
Absorption correction: multi-scan (CrysAlisPro; Rigaku OD, 2020)	*h* = −14→15
*T*_min_ = 0.519, *T*_max_ = 1.000	*k* = −21→22
36223 measured reflections	*l* = −13→13

### Refinement

**Table:**

Refinement on *F*^2^	Hydrogen site location: mixed
Least-squares matrix: full	H atoms treated by a mixture of independent and constrained refinement
*R*[*F*^2^ > 2σ(*F*^2^)] = 0.026	*w* = 1/[σ^2^(*F*_o_^2^) + (0.0473*P*)^2^ + 1.8799*P*] where *P* = (*F*_o_^2^ + 2*F*_c_^2^)/3
*wR*(*F*^2^) = 0.075	(Δ/σ)_max_ < 0.001
*S* = 1.20	Δρ_max_ = 0.72 e Å^−^^3^
2475 reflections	Δρ_min_ = −0.99 e Å^−^^3^
182 parameters	Extinction correction: SHELXL (Sheldrick, 2015), Fc^*^=kFc[1+0.001xFc^2^λ^3^/sin(2θ)]^-1/4^
1 restraint	Extinction coefficient: 0.00067 (9)
Primary atom site location: structure-invariant direct methods	Absolute structure: Flack *x* determined using 1131 quotients [(*I*^+^)-(*I*^-^)]/[(*I*^+^)+(*I*^-^)] (Parsons *et al.*, 2013).
Secondary atom site location: difference Fourier map	Absolute structure parameter: −0.012 (4)

### Special details

**Table:**

Geometry. All esds (except the esd in the dihedral angle between two l.s. planes) are estimated using the full covariance matrix. The cell esds are taken into account individually in the estimation of esds in distances, angles and torsion angles; correlations between esds in cell parameters are only used when they are defined by crystal symmetry. An approximate (isotropic) treatment of cell esds is used for estimating esds involving l.s. planes.

### Fractional atomic coordinates and isotropic or equivalent isotropic displacement parameters (Å^2^)

**Table:**

	*x*	*y*	*z*	*U*_iso_*/*U*_eq_	
C1	0.6224 (3)	0.6694 (2)	0.9539 (4)	0.0255 (8)	
C2	0.7242 (3)	0.6887 (2)	0.9964 (4)	0.0322 (9)	
C3	0.7340 (5)	0.7364 (3)	1.0986 (5)	0.0422 (13)	
H3	0.802253	0.749817	1.127537	0.051*	
C4	0.6462 (5)	0.7642 (2)	1.1578 (4)	0.0439 (12)	
H4	0.654329	0.795728	1.227906	0.053*	
C5	0.5452 (5)	0.7465 (2)	1.1153 (4)	0.0399 (11)	
H5	0.484590	0.766539	1.155165	0.048*	
C6	0.5341 (4)	0.6995 (2)	1.0149 (4)	0.0316 (8)	
H6	0.465320	0.687281	0.986326	0.038*	
C7	0.6043 (3)	0.61456 (19)	0.8526 (3)	0.0220 (7)	
C8	0.7448 (4)	0.5100 (2)	0.6398 (5)	0.0240 (9)	
C9	0.7096 (3)	0.4582 (2)	0.5418 (4)	0.0247 (8)	
C10	0.7783 (3)	0.4270 (2)	0.4540 (4)	0.0319 (9)	
H10	0.851195	0.439329	0.458193	0.038*	
C11	0.6425 (4)	0.3646 (2)	0.3636 (4)	0.0365 (10)	
H11	0.616344	0.331030	0.302482	0.044*	
C12	0.5728 (3)	0.3950 (2)	0.4482 (4)	0.0282 (8)	
H12	0.500036	0.382194	0.443503	0.034*	
C13	0.8597 (3)	0.5252 (3)	0.6647 (5)	0.0388 (11)	
H13A	0.879522	0.502577	0.743598	0.058*	
H13B	0.871828	0.580037	0.667603	0.058*	
H13C	0.902554	0.502866	0.599080	0.058*	
Cd1	0.500000	0.500000	0.68642 (13)	0.01811 (16)	
N1	0.6931 (3)	0.58932 (17)	0.7964 (3)	0.0236 (6)	
N2	0.6699 (2)	0.54015 (16)	0.7035 (3)	0.0211 (6)	
N3	0.6056 (2)	0.44157 (17)	0.5356 (3)	0.0227 (6)	
N4	0.7454 (4)	0.3809 (2)	0.3652 (4)	0.0398 (9)	
O1	0.8136 (3)	0.66287 (19)	0.9425 (3)	0.0393 (7)	
H1	0.796 (6)	0.631 (4)	0.880 (7)	0.059*	
O2	0.5115 (2)	0.5951 (2)	0.8272 (4)	0.0271 (8)	

### Atomic displacement parameters (Å^2^)

**Table:**

	*U*^11^	*U*^22^	*U*^33^	*U*^12^	*U*^13^	*U*^23^
C1	0.031 (2)	0.0204 (17)	0.0249 (18)	−0.0051 (13)	−0.0068 (15)	0.0044 (15)
C2	0.039 (2)	0.0289 (18)	0.0291 (19)	−0.0115 (17)	−0.0106 (18)	0.0059 (16)
C3	0.062 (3)	0.0319 (18)	0.032 (2)	−0.019 (2)	−0.020 (2)	0.004 (2)
C4	0.076 (4)	0.029 (2)	0.026 (2)	−0.015 (2)	−0.010 (2)	−0.0013 (17)
C5	0.064 (4)	0.025 (2)	0.030 (2)	−0.010 (2)	0.000 (2)	−0.0017 (18)
C6	0.041 (2)	0.0249 (19)	0.028 (2)	−0.0056 (19)	−0.003 (2)	−0.0002 (16)
C7	0.0221 (17)	0.0225 (16)	0.0214 (16)	−0.0039 (13)	−0.0030 (14)	0.0033 (14)
C8	0.014 (2)	0.0278 (18)	0.031 (2)	0.0012 (15)	0.0002 (18)	0.0100 (16)
C9	0.0225 (19)	0.0237 (16)	0.0279 (18)	0.0048 (13)	0.0074 (15)	0.0079 (14)
C10	0.030 (2)	0.0273 (18)	0.038 (2)	0.0084 (15)	0.0141 (17)	0.0076 (17)
C11	0.055 (3)	0.0276 (19)	0.027 (2)	0.0030 (19)	0.010 (2)	−0.0020 (16)
C12	0.033 (2)	0.0258 (17)	0.0261 (17)	−0.0022 (15)	0.0027 (16)	0.0024 (16)
C13	0.0136 (18)	0.045 (2)	0.058 (3)	−0.0028 (18)	0.0024 (19)	0.010 (3)
Cd1	0.0110 (2)	0.0243 (2)	0.0190 (2)	−0.00150 (9)	0.000	0.000
N1	0.0208 (15)	0.0243 (15)	0.0256 (15)	−0.0053 (12)	−0.0046 (12)	0.0016 (12)
N2	0.0155 (14)	0.0234 (12)	0.0243 (16)	−0.0022 (11)	0.0008 (12)	0.0048 (12)
N3	0.0207 (15)	0.0243 (14)	0.0230 (13)	0.0016 (11)	0.0039 (12)	0.0029 (12)
N4	0.049 (2)	0.0302 (17)	0.040 (2)	0.0100 (17)	0.0206 (18)	0.0047 (16)
O1	0.0320 (17)	0.0460 (17)	0.0399 (16)	−0.0136 (13)	−0.0121 (14)	0.0016 (15)
O2	0.0208 (14)	0.0311 (17)	0.0293 (18)	−0.0023 (10)	−0.0010 (11)	−0.0091 (15)

### Geometric parameters (Å, º)

**Table:**

C1—C6	1.405 (7)	C10—N4	1.330 (7)
C1—C2	1.412 (6)	C10—H10	0.9500
C1—C7	1.485 (5)	C11—N4	1.335 (7)
C2—O1	1.354 (6)	C11—C12	1.385 (6)
C2—C3	1.401 (7)	C11—H11	0.9500
C3—C4	1.375 (8)	C12—N3	1.324 (5)
C3—H3	0.9500	C12—H12	0.9500
C4—C5	1.396 (8)	C13—H13A	0.9800
C4—H4	0.9500	C13—H13B	0.9800
C5—C6	1.378 (6)	C13—H13C	0.9800
C5—H5	0.9500	Cd1—N2	2.273 (3)
C6—H6	0.9500	Cd1—N2^i^	2.273 (3)
C7—O2	1.255 (5)	Cd1—O2	2.277 (4)
C7—N1	1.355 (5)	Cd1—O2^i^	2.277 (4)
C8—N2	1.289 (6)	Cd1—N3^i^	2.356 (3)
C8—C9	1.475 (7)	Cd1—N3	2.356 (3)
C8—C13	1.504 (7)	N1—N2	1.365 (4)
C9—N3	1.351 (5)	O1—H1	0.90 (8)
C9—C10	1.405 (5)		
			
C6—C1—C2	118.7 (4)	N3—C12—H12	119.4
C6—C1—C7	118.4 (4)	C11—C12—H12	119.4
C2—C1—C7	122.8 (4)	C8—C13—H13A	109.5
O1—C2—C3	118.2 (4)	C8—C13—H13B	109.5
O1—C2—C1	122.7 (4)	H13A—C13—H13B	109.5
C3—C2—C1	119.1 (5)	C8—C13—H13C	109.5
C4—C3—C2	121.0 (5)	H13A—C13—H13C	109.5
C4—C3—H3	119.5	H13B—C13—H13C	109.5
C2—C3—H3	119.5	N2—Cd1—N2^i^	170.63 (17)
C3—C4—C5	120.4 (4)	N2—Cd1—O2	69.83 (11)
C3—C4—H4	119.8	N2^i^—Cd1—O2	103.58 (11)
C5—C4—H4	119.8	N2—Cd1—O2^i^	103.58 (11)
C6—C5—C4	119.4 (5)	N2^i^—Cd1—O2^i^	69.83 (11)
C6—C5—H5	120.3	O2—Cd1—O2^i^	95.4 (2)
C4—C5—H5	120.3	N2—Cd1—N3^i^	117.27 (11)
C5—C6—C1	121.4 (5)	N2^i^—Cd1—N3^i^	69.86 (11)
C5—C6—H6	119.3	O2—Cd1—N3^i^	100.55 (13)
C1—C6—H6	119.3	O2^i^—Cd1—N3^i^	139.07 (10)
O2—C7—N1	126.0 (3)	N2—Cd1—N3	69.86 (11)
O2—C7—C1	119.0 (4)	N2^i^—Cd1—N3	117.27 (11)
N1—C7—C1	114.9 (3)	O2—Cd1—N3	139.07 (10)
N2—C8—C9	115.0 (4)	O2^i^—Cd1—N3	100.55 (13)
N2—C8—C13	122.8 (5)	N3^i^—Cd1—N3	91.59 (16)
C9—C8—C13	122.2 (4)	C7—N1—N2	111.4 (3)
N3—C9—C10	119.0 (4)	C8—N2—N1	120.2 (3)
N3—C9—C8	117.7 (4)	C8—N2—Cd1	121.6 (3)
C10—C9—C8	123.2 (4)	N1—N2—Cd1	117.5 (2)
N4—C10—C9	122.7 (4)	C12—N3—C9	118.5 (3)
N4—C10—H10	118.6	C12—N3—Cd1	126.5 (3)
C9—C10—H10	118.6	C9—N3—Cd1	115.1 (3)
N4—C11—C12	122.1 (4)	C10—N4—C11	116.5 (4)
N4—C11—H11	119.0	C2—O1—H1	109 (5)
C12—C11—H11	119.0	C7—O2—Cd1	114.1 (3)
N3—C12—C11	121.2 (4)		
			
C6—C1—C2—O1	−179.3 (4)	C8—C9—C10—N4	−179.3 (4)
C7—C1—C2—O1	4.8 (6)	N4—C11—C12—N3	−0.4 (6)
C6—C1—C2—C3	0.8 (6)	O2—C7—N1—N2	1.7 (5)
C7—C1—C2—C3	−175.2 (4)	C1—C7—N1—N2	−179.1 (3)
O1—C2—C3—C4	−179.7 (4)	C9—C8—N2—N1	−179.8 (3)
C1—C2—C3—C4	0.2 (6)	C13—C8—N2—N1	1.4 (6)
C2—C3—C4—C5	−1.3 (7)	C9—C8—N2—Cd1	10.1 (5)
C3—C4—C5—C6	1.3 (6)	C13—C8—N2—Cd1	−168.8 (3)
C4—C5—C6—C1	−0.3 (6)	C7—N1—N2—C8	−179.7 (3)
C2—C1—C6—C5	−0.7 (6)	C7—N1—N2—Cd1	−9.2 (4)
C7—C1—C6—C5	175.4 (4)	C11—C12—N3—C9	−1.0 (6)
C6—C1—C7—O2	−2.2 (5)	C11—C12—N3—Cd1	179.4 (3)
C2—C1—C7—O2	173.7 (4)	C10—C9—N3—C12	1.5 (5)
C6—C1—C7—N1	178.6 (3)	C8—C9—N3—C12	−179.9 (3)
C2—C1—C7—N1	−5.5 (5)	C10—C9—N3—Cd1	−178.8 (3)
N2—C8—C9—N3	−6.2 (5)	C8—C9—N3—Cd1	−0.2 (4)
C13—C8—C9—N3	172.6 (4)	C9—C10—N4—C11	−0.6 (6)
N2—C8—C9—C10	172.3 (4)	C12—C11—N4—C10	1.2 (6)
C13—C8—C9—C10	−8.8 (6)	N1—C7—O2—Cd1	6.4 (5)
N3—C9—C10—N4	−0.8 (6)	C1—C7—O2—Cd1	−172.7 (3)

Symmetry code: (i) −*x*+1, −*y*+1, *z*.

### Hydrogen-bond geometry (Å, º)

*Cg*1 and *Cg*2 are the centroids of the C1–C6 and N3–N4/C9–C12 rings, respectively.

**Table:**

*D*—H···*A*	*D*—H	H···*A*	*D*···*A*	*D*—H···*A*
C4—H4···N4^ii^	0.95	2.47	3.349 (6)	154
C10—H10···O2^iii^	0.95	2.55	3.283 (5)	134
C12—H12···O1^iv^	0.95	2.49	3.439 (5)	174
O1—H1···N1	0.90 (8)	1.76 (8)	2.557 (4)	146 (7)
C13—H13*A*···*Cg*2^v^	0.98	2.86	3.740 (6)	149
C13—H13*B*···*Cg*1^vi^	0.98	2.71	3.592 (6)	150

Symmetry codes: (ii) −*x*+3/2, *y*+1/2, *z*+1; (iii) *x*+1/2, −*y*+1, *z*−1/2; (iv) *x*−1/2, −*y*+1, *z*−1/2; (v) −*x*+3/2, *y*, *z*+1/2; (vi) −*x*+3/2, *y*, *z*−1/2.

## References

[bb1] Abboud, K. A., Palenik, R. C., Palenik, G. J. & Wood, R. M. (2007). *Inorg. Chim. Acta*, **360**, 3642–3646.

[bb2] Barbazán, P., Carballo, R. & Vázquez-López, E. M. (2007). *CrystEngComm*, **9**, 668–675.

[bb3] Brines, L. M., Shearer, J., Fender, J. K., Schweitzer, D., Shoner, S. C., Barnhart, D., Kaminsky, W., Lovell, S. & Kovacs, J. A. (2007). *Inorg. Chem.* **46**, 9267–9277. 10.1021/ic701433p PMC2532082 17867686

[bb4] Cindrić, M., Bjelopetrović, A., Pavlović, G., Damjanović, V., Lovrić, J., Matković-Čalogović, D. & Vrdoljak, V. (2017). *New J. Chem.* **41**, 2425–2435.

[bb5] Dang, D.-B., Bai, Y. & Duan, C.-Y. (2006*a*). *Acta Cryst.* E**62**, m1567–m1568.

[bb6] Dang, D.-B., Bai, Y. & Duan, C.-Y. (2006*b*). *Acta Cryst.* E**62**, m2290–m2292.

[bb7] Database of Ionic Radii (2020). Hosted by the Atomistic Simulation Group in the Materials Department of Imperial College, http://abulafia.mt.ic.ac.uk/shannon/*PTABLE*.php

[bb8] Groom, C. R., Bruno, I. J., Lightfoot, M. P. & Ward, S. C. (2016). *Acta Cryst.* B**72**, 171–179. 10.1107/S2052520616003954 PMC4822653 27048719

[bb9] Huang, W., Shen, F.-X., Wu, S.-Q., Liu, L., Wu, D., Zheng, Z., Xu, J., Zhang, M., Huang, X.-C., Jiang, J., Pan, F., Li, Y., Zhu, K. & Sato, O. (2016). *Inorg. Chem.* **55**, 5476–5484. 10.1021/acs.inorgchem.6b00500 27164298

[bb10] Kalinowski, D. S., Sharpe, P. C., Bernhardt, P. V. & Richardson, D. R. (2008). *J. Med. Chem.* **51**, 331–344. 10.1021/jm7012562 18159922

[bb11] Kar, C., Samanta, S., Goswami, S., Ramesh, A. & Das, G. (2015). *Dalton Trans.* **44**, 4123–4132. 10.1039/c4dt01433b 25622931

[bb12] Kuriakose, D., Aravindakshan, A. A. & Kurup, M. R. P. (2017). *Polyhedron*, **127**, 84–96.

[bb13] Manna, A. K., Chowdhury, S. & Patra, G. K. (2019). *Dalton Trans.* **48**, 12336–12348. 10.1039/c9dt02448d 31347648

[bb14] Mondal, S., Naskar, S., Dey, A. K., Sinn, E., Eribal, C., Herron, S. R. & Chattopadhyay, S. K. (2013). *Inorg. Chim. Acta*, **398**, 98–105.

[bb15] Parsons, S., Flack, H. D. & Wagner, T. (2013). *Acta Cryst.* B**69**, 249–259. 10.1107/S2052519213010014 PMC3661305 23719469

[bb16] Patel, R. N., Singh, Y., Singh, Y. P., Patel, A. K., Patel, N., Singh, R., Butcher, R. J., Jasinski, J. P., Colacio, E. & Palacios, M. A. (2018). *New J. Chem.* **42**, 3112–3136.

[bb17] Reger, D. L., Pascui, A. E., Smith, M. D., Jezierska, J. & Ozarowski, A. (2012). *Inorg. Chem.* **51**, 11820–11836. 10.1021/ic301757g 23043562

[bb18] Rigaku OD (2020). *CrysAlis PRO*. Rigaku Oxford Diffraction, Yarnton, England.

[bb19] Sen, S., Talukder, P., Rosair, G. & Mitra, S. (2005). *Struct. Chem.* **16**, 605–610.

[bb20] Sheldrick, G. M. (2008). *Acta Cryst.* A**64**, 112–122. 10.1107/S0108767307043930 18156677

[bb21] Sheldrick, G. M. (2015). *Acta Cryst.* C**71**, 3–8.

[bb22] Shit, S., Chakraborty, J., Samanta, B., Slawin, A. M. Z., Gramlich, V. & Mitra, S. (2009). *Struct. Chem.* **20**, 633–642.

[bb23] Sola, M., Mestres, J., Duran, M. & Carbo, R. (1994). *J. Chem. Inf. Comput. Sci.* **34**, 1047–1053.

[bb24] Sousa-Pedrares, A., Camiña, N., Romero, J., Durán, M. L., García-Vázquez, J. A. & Sousa, A. (2008). *Polyhedron*, **27**, 3391–3397.

[bb25] Sutradhar, M., Kirillova, M. V., Guedes da Silva, M. F. C., Liu, C.-M. & Pombeiro, A. J. L. (2013). *Dalton Trans.* **42**, 16578–16587. 10.1039/c3dt52453a 24068161

[bb26] Tai, X.-S. & Feng, Y.-M. (2008). *Acta Cryst.* E**64**, o707–o707.

[bb27] Tai, X.-S., Feng, Y.-M. & Zhang, H.-X. (2008). *Acta Cryst.* E**64**, m656. 10.1107/S1600536808009707 PMC2961272 21202202

[bb28] Westrip, S. P. (2010). *J. Appl. Cryst.* **43**, 920–925.

[bb29] Xu, S.-P., Yang, F.-L., Zhu, G.-Z., Shi, H.-L. & Li, X.-L. (2014). *Polyhedron*, **68**, 1–9.

[bb30] Yang, P. (2019). CSD Communications (refcodes CIZJED, CIZGOK and CIZFUP). CCDC, Cambridge, England.

[bb31] Yang, P., Chen, H., Wang, Z.-Z., Zhang, L.-L., Zhang, D.-D., Shi, Q.-S. & Xie, X.-B. (2020). *J. Inorg. Biochem.* **213**, 111248–111248. 10.1016/j.jinorgbio.2020.111248 33011623

[bb32] Yang, P., Zhang, D.-D., Wang, Z.-Z., Liu, H.-Z., Shi, Q.-S. & Xie, X.-B. (2019). *Dalton Trans.* **48**, 17925–17935. 10.1039/c9dt03746b 31793567

[bb33] Yang, P., Zhang, L.-L., Wang, Z.-Z., Zhang, D.-D., Liu, Y.-M., Shi, Q.-S. & Xie, X.-B. (2020). *J. Inorg. Biochem.* **203**, 110919–110919. 10.1016/j.jinorgbio.2019.110919 31783217

[bb34] You, Z., Yu, H., Li, Z., Zhai, W., Jiang, Y., Li, A., Guo, S., Li, K., Lv, C. & Zhang, C. (2018). *Inorg. Chim. Acta*, **480**, 120–126.

[bb35] Zhang, L., Xu, G.-C., Xu, H.-B., Mereacre, V., Wang, Z.-M., Powell, A. K. & Gao, S. (2010). *Dalton Trans.* **39**, 4856–4868. 10.1039/b925482j 21491699

